# Effects of a novel variant of the yeast γ-glutamyl kinase Pro1 on its enzymatic activity and sake brewing

**DOI:** 10.1007/s10295-020-02297-1

**Published:** 2020-08-03

**Authors:** Naoyuki Murakami, Atsushi Kotaka, Shota Isogai, Keiko Ashida, Akira Nishimura, Kengo Matsumura, Yoji Hata, Hiroki Ishida, Hiroshi Takagi

**Affiliations:** 1 Research Institute, Gekkeikan Sake Co. Ltd., 101 Shimotoba-koyanagi-cho, Fushimi-ku, Kyoto 612-8385 Japan; 2 Graduate School of Science and Technology, Nara Institute of Science and Technology, 8916-5 Takayama, Ikoma, Nara 630-0192 Japan

**Keywords:** γ-Glutamyl kinase Pro1, Proline, Succinate, *Saccharomyces cerevisiae*, Sake yeast

## Abstract

Sake is a traditional Japanese alcoholic beverage brewed with the yeast *Saccharomyces cerevisiae*. Sake taste is affected by sugars, organic acids, and amino acids. We previously isolated mutants resistant to the proline analogue azetidine-2-carboxylate derived from a diploid sake yeast strain. Some of the mutants produced a greater amount of proline in the brewed sake. One of them (strain K-9-AZC) carried a novel mutation in the *PRO1* gene encoding the Gln79His variant of the γ-glutamyl kinase Pro1, a key enzyme in proline biosynthesis in *S. cerevisiae*. This mutation resulted in extreme desensitization to feedback inhibition by proline, leading to proline overproduction. Interestingly, sake brewed with K-9-AZC contained 3.7-fold more proline, but only 25% less succinate than sake brewed with the parent strain. Metabolome analysis suggests that the decrease in succinate was attributable to a lower level of 2-oxoglutarate, which is converted into glutamate. The approach here could be a practical method for breeding of yeast strains involved in the diversity of sake taste.

## Introduction

Sake is a traditional Japanese alcoholic beverage made from steamed rice by multiple parallel fermentations of the fungus *Aspergillus oryzae* and the yeast *Saccharomyces cerevisiae*, which produce saccharification enzymes and ethanol from glucose, respectively. The taste of sake is determined by a combination of many compounds such as sugars, organic acids, amino acids, nucleotides, and inorganic salts. In particular, since sugars are sweet and organic acids are sour, it is possible to make sake of various tastes by changing the balance of sugars and organic acids.

On the other hand, amino acids with sweet, bitter, or umami (the pleasant savory taste of foods, such as seaweed, cured fish, aged cheeses, and meats, elicited by glutamate) taste may impact some sensory qualities of sake, but too much amino acid content is often thought to produce an unfavorable taste in sake. In sake mash, amino acids are derived mainly from the digestion of rice proteins by sake *koji* enzymes; however, yeast cells also synthesize them during fermentation. In yeast, amino acid metabolism and its regulatory mechanisms vary under different growth environments by regulating anabolic and catabolic processes, including uptake and export, and the metabolic styles form a complicated and robust network. There is also crosstalk with various metabolic pathways, products, and signal molecules. The elucidation of metabolic regulatory mechanisms and physiological roles is important fundamental research for understanding life phenomena [8, 21]. However, a lack of knowledge concerning the mechanism underlying amino acid production during sake fermentation has made it difficult to develop yeast strains with different amino acid profiles. The development of strains that can produce specific or various amino acids could enable the production of sake with distinctive tastes.

In sake, not only amino acids but also other taste components originate mainly from *S. cerevisiae* cells, and a variety of yeast strains have been constructed to develop differentiated sake products [6]. Recently, we bred a diploid sake yeast strain K-9-AZC that produced higher proline and lower succinate levels than those of its parent strain Kyokai no. 9 (K-9) in sake mash, leading to the potential for low-carbohydrate sake [7]. From strain K-9, many mutants including strain K-9-AZC were isolated from minimal agar medium containing 500 mg/l of toxic proline analogue azetidine-2-carboxylic acid (AZC) by spontaneous mutagenesis [7]. In general, microorganisms that overproduce various amino acids have been obtained by isolating mutants resistant to analogues of corresponding amino acids. Some AZC-resistant mutants were found to accumulate larger amounts of intracellular proline than the parent strain. Proline-accumulating *S. cerevisiae* strains usually have a mutation on the *PRO1* gene, which encodes γ-glutamyl kinase (GK). GK is the rate-limiting enzyme of proline biosynthesis from glutamate, and its activity is regulated allosterically by the end product proline [15]. The majority of proline-accumulating strains have been obtained by expressing GK variants, such as Asp154Asn (D154N) and Ile150Thr (I150T), which are less sensitive to feedback inhibition by proline [10, 15].

However, the molecular mechanisms involved in both higher proline and lower succinate production in the AZC-resistant mutant K-9-AZC have not been yet clarified. In the present study, we identified a novel mutation in the *PRO1* gene encoding, the Gln79His variant of GK (Q79H), which increases intracellular proline levels, in strain K-9-AZC. We also analyzed the fermentation and metabolite profiles of strain K-9-AZC during sake brewing.

## Materials and methods

### Strains and media

We used the diploid Japanese sake yeast strain Kyokai no. 9 (K-9) of *S. cerevisiae*. Strain K-9-AZC, which is the AZC-resistant mutant, was obtained from strain K-9 by spontaneous mutagenesis [7]. Yeast cells were cultured in a nutrient-rich medium YPD (2% glucose, 1% yeast extract, and 2% peptone) and a synthetic minimal medium SD (2% glucose and 0.67% yeast nitrogen base) without amino acids (Becton, Dickinson and Company).

*Escherichia coli* strains DH5α (F^−^λ^−^Φ*80lacZ*Δ*M15* Δ(*lacZYA argF*)*U169 deoR recA1 endA1 hsdR17*(*r*_*k*_^−^*m*_*k*_^+^) *supE44 thi*-*1 gyrA96*) and BL21 (DE3) (F^−^ *ompT hsdS*(r_B_^−^ m_B_^−^) *gal dcm λ*(DE3) (λ(DE3):*lacI*, *lacUV5*-*T7 gene1 ind1 sam7 nin5*) were used for cloning of the gene and for expression of the recombinant GK, respectively. *E. coli* strains were cultured in Luria–Bertani (LB) (0.5% yeast extract, 1% tryptone and 1% NaCl) containing 100 μg/ml ampicillin or M9CA medium (0.4% glucose, 2% casamino acid, 65 mM sodium/potassium phosphate, 8.6 mM NaCl, 18.7 mM ammonium chloride, 1 mM MgSO_4_, and 0.1 mM CaCl_2_) containing 100 μg/ml ampicillin.

### Cloning and sequencing of the *PRO1* gene

The full-length *PRO1* gene flanked by 500 bp upstream and downstream of the open reading frame was amplified from the genomic DNA of K-9 and K-9-AZC by PCR using KOD FX Neo DNA polymerase (Toyobo) using primers PRO1-0 and PRO1-5 (Table 1). To confirm the introduced mutations, the PCR products were sequenced by DNA sequencing. The *PRO1* gene DNA sequences of sake yeast mutants were compared to that of the sake yeast strain *S. cerevisiae* Kyokai no. 7 (K-7) using the BLAST search program. The GeneBank accession number for the *PRO1* gene is M85293.

**Table 1:** Primers used in this study

Primer	Sequence (5′→3′)	Description
PRO1-0	CAG TGA AGT GTT CAA GGG	For gene cloning
PRO1-5	CTT CCA AGG GTA GGA AA	
PRO1_pET_fw	CGC GCG GCA GCC ATA TGA AGG ATG CTA ATG AGA G	
PRO1_pET_rv	CAG CCG GAT CCT CGA GTC AAC GAG GTG GGA ATG C	
PRO1_I150T_fw	GTT AGA GAA AC*C AAA TTT GGT GAC AAT GAC	For site-directed mutagenesis
PRO1_I150T_rv	ACC AAA TTT GG*T TTC TCT AAC AGA TAG TGT	

The asterisks indicate the positions of the nucleotide mutation

### Construction of expression plasmids for the *PRO1* gene

The wild-type and Q79H mutant *PRO1* genes were amplified from the genomic DNA of K-9 and K-9-AZC, respectively, by PCR with the primers PRO1_pET_fw and PRO1_pET_rv (Table 1). The PCR-amplified DNA fragments were introduced into the *Nde*I/*Xho*I site of vector pET15b (Novagen) using In Fusion^®^ HD Cloning Kit (Takara Bio), resulting in plasmids pET-*PRO1* (WT) and pET-*PRO1* (Q79H). The point mutation (T to C at position 449), leading to the I150T substitution in GK [15], was introduced into the *PRO1* gene on pET15b by PCR with the primers, PRO1_I150T_fw and PRO1_I150T_rv (Table 1), generated plasmid pET-*PRO1* (I150T). The nucleotide sequences of the *PRO1* genes were verified and all expression plasmids were introduced into *E. coli* BL21 (DE3) cells.

### Expression and purification of the recombinant GKs

*Escherichia coli* BL21 (DE3) cells harboring pET-*PRO1* (WT), pET-*PRO1* (Q79H), and pET-*PRO1* (I150T) were cultivated in 100 ml of M9CA medium containing ampicillin and grown at 37 °C to an optical density 600 nm (OD_600_) of 0.8. The cells were cooled on ice for 5 min and isopropyl β-d-1-thiogalactopyranoside (IPTG) was added to a final concentration of 0.5 mM. After 20 h of cultivation at 18 °C, the cells were harvested by centrifugation and suspended in 10 ml of buffer A (50 mM Tris–HCl (pH7.4), 150 mM NaCl, and 20% (w/v) glycerol). The cell suspension was homogenized under cooling, and then centrifuged for 20 min at 8000 rpm at 4 °C. The supernatant was applied onto a nickel affinity column (Ni Sepharose^TM^ 6 Fast flow, GE Healthcare Life Sciences). After the column was washed with buffer A containing 100 mM imidazole, the recombinant proteins were eluted by buffer A supplemented with 500 mM imidazole.

### Assay of GK activity

GK activity was measured by the production of ADP in an enzyme-coupled system with pyruvate kinase (PK) and lactate dehydrogenase (LDH) [16, 25]. The reaction mixture (final volume, 1 ml) contained the following: 100 mM HEPES–NaOH pH 7.4, 400 mM sodium glutamate, 5 mM ATP, 10 mM MgCl_2_, 1 mM phosphoenoylpyruvate, 0.25 mM NADH, 7.5 U PK/LDH (Sigma-Aldrich), and 2 μg of purified GK. The reaction mixture was pre-equilibrated for 3 min at 30 °C, and then the reaction was initiated by the addition of sodium glutamate. GK-dependent oxidation of NADH was monitored at 340 nm with a DU-800 spectrophotometer (Beckman Coulter) and maintained at 30 °C. To examine the feedback inhibition sensitivity of GKs, proline was added to the reaction mixture at a concentration of 0–5 mM for WT and 0–100 mM for Q79H and I150T. The reaction rate was calculated with the extinction coefficient of NADH, 6220 M^−1^ cm^−1^. One unit of activity was defined as the amount of enzyme required to produce 1 μmol of ADP per min.

### One-step sake brewing test

Yeasts were cultivated in YPD medium at 30 °C for 48 h with shaking at 120 rpm, harvested by centrifugation, and resuspended in water. Sake brewing test was carried out according to previously reported method [11], using 136 g of polished rice with a polishing ratio of 78%, 34 g of rice *koji* (*A. oryzae* was grown on steamed rice), 80 µl 90% lactic acid, 425 ml of water, and sake yeast cells were inoculated into sake mash to 1 × 10^7^ cells per ml of sake mash. To separate the yeasts from sake mash, rice grains were homogenized, gelatinized, and digested to oligosaccharide with a thermostable α-amylase Kleistase (Amano Enzyme). Fermentation was carried out at 15 °C. Fermentation was monitored by measuring the weight of sake mash, which represents CO_2_ evolution. After 9 days of fermentation at 15 °C (CO_2_ emission was reached at 380 g/kg total rice weight), sake mash was centrifuged and supernatant was obtained as sake.

### Measurement of supernatant metabolites of sake mash

We measured the supernatant metabolites of sake mash. The ethanol concentration was measured after supernatant was distilled using Density/Specific Gravity Meter DA-650 (Kyoto Electronics Manufacturing). Acid and amino acid contents were measured with an electric potential difference autotitration apparatus (Kyoto Electronics Manufacturing) by the National Tax Administration Agency method [12]. Organic acids and amino acids concentrations were analyzed using HPLC Organic Acid Analyser and Ultra HPLC Amino Acid Analyser (Shimadzu), respectively.

### Measurement of intracellular metabolites of sake mash

Cell samples harvested from sake mash. First, 10 ml sake mash was filtrated at 100 mesh filter. The filtration and centrifuged (7000×*g*, 4 °C, 5 min), the supernatant was stored at − 20 °C until measuring the extracellular metabolites. Besides, removed the starch paste surface of the sake lees completely, and suspended with dilution water. In addition, the suspension was sampled 1.0 ml in 3 or 4 tubes and spin down at the max speed only a brief moment (< 1 s) to used tabletop centrifuge NSD-12 (Nissinrika), not completely separate solid and liquid, cloudy supernatant was transferred with 500 μl to new tube. Once again centrifuged at the max speed (> 15 s) and removed the supernatant completely, additionally suspended all tube’s pellet with dilution water at 1.0 ml and adjusted to an OD_600_ of 10.0. Then, centrifuged (7000×*g*, 4 °C, 5 min) and completely removed the supernatant and the pellet was suspended with 1.6 ml methanol completely and add the 1.1 ml of Milli-Q water containing internal standards (Solution ID: H3304-1002, Human Metabolome Technologies) and mixed thoroughly. The mixture was centrifugally filtered through a Millipore 5-kDa cutoff filter at 9100×*g* and 4 °C for 5 h to remove proteins and macromolecules. Finally, the intracellular extracted samples were stored at − 80 °C until measuring the intracellular metabolites.

Metabolome measurements were carried out through a facility service at Human Metabolome Technologies. CE-TOFMS was carried out using an Agilent CE Capillary Electrophoresis System equipped with an Agilent 6210 Time of Flight mass spectrometer, Agilent 1100 isocratic HPLC pump, Agilent G1603A CE-MS adapter kit, and Agilent G1607A CE-ESI–MS sprayer kit (Agilent Technologies). The systems were controlled by Agilent G2201AA ChemStation software version B.03.01 for CE (Agilent Technologies). The metabolites were analyzed using a fused silica capillary (50 μm *i.d. *× 80 cm total length), with commercial electrophoresis buffer (Solution ID: H3301-1001 for cation analysis and H3302-1023 for anion analysis, Human Metabolome Technologies) as the electrolyte. The sample was injected at a pressure of 50 mbar for 10 s (approximately 10 nl) in cation analysis and 25 s (approximately 25 nl) in anion analysis. The spectrometer was scanned from *m/z* 50 to 1000. Other conditions were as in the described previously [17–19]. Peaks were extracted using automatic integration software MasterHands (Keio University) to obtain peak information including *m/z*, migration time for CE-TOFMS measurement (MT) and peak area [20]. Signal peaks corresponding to isotopomers, adduct ions, and other product ions of known metabolites were excluded, and remaining peaks were annotated with putative metabolites from the HMT metabolite database based on their MTs and *m/z* values determined by TOFMS. The tolerance range for the peak annotation was configured at ± 0.5 min for MT and ± 10 ppm for *m/z*. In addition, peak areas were normalized against those of the internal standards and then the resultant relative area values were further normalized by sample amount. Hierarchical cluster analysis (HCA) was performed by HMT proprietary software, PeakStat.

## Results and discussion

### Nucleotide sequence of the *PRO1* gene in K-9-AZC

**Fig. 1:**
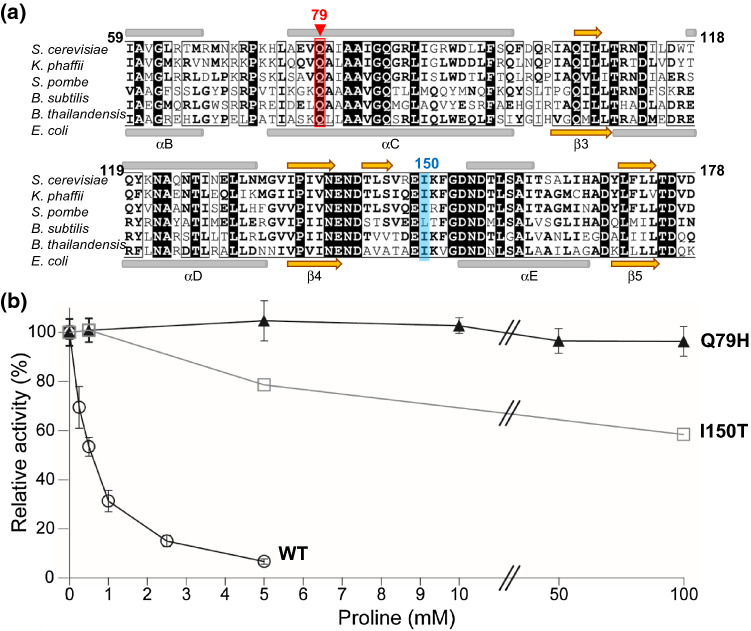
**a** Partial amino acid sequence alignment of GKs among various microorganisms. The amino acid sequence of the *S. cerevisiae* GK was compared to *Komagataella phaffii* (UniProt ID code: F2QWU5), *Schizosaccharomyces pombe* (O13810), *Bacillus subtilis* (P39820), *Burkholderia thailandensis* (Q2SZF9), and *Escherichia coli* (P0A7B5) homologues. Numbering of residues is in ScGK and conserved residues were highlighted in black boxes. Gln79 and Ile150 are shown in red and light blue, respectively. Gray bars and orange arrows above the alignment represented the hypothetical α-helices and β-sheets of ScGK, predicted by Jpred 4 (A Protein Secondary Structure Prediction Server, http://www.compbio.dundee.ac.uk/jpred4/); those under the alignment were the secondary structure in the crystal structure of EcGK (PDB ID code:2J5T) [9]. **b** Effect of proline on GK activity. The GK activities of the wild-type (open circle), Q79H (filled triangle), and I150T (open square) variant GKs were measured in the presence of proline. The relative activities are expressed corresponding to the parameters in the absence of proline. The values for the wild-type and Q79H variant GKs are the means and standard deviations of results from three independent experiments. The value for the I150T variant GK was obtained from one experiment

Many proline-accumulating mutant strains [7] were confirmed to have mutations in the *PRO1* gene encoding GK (such as G460→A and T449→C), which is a key enzyme in proline biosynthesis in *S. cerevisiae* [5, 10, 15, 24]. To identify the mutations that confer AZC resistance and proline accumulation, we analyzed the nucleotide sequences of the *PRO1* genes from parent K-9 and mutant K-9-AZC strains using direct PCR DNA sequencing and compared them to that of the *S. cerevisiae* sake yeast strain K-7 using the BLAST search program. The results showed that the sequence of the *PRO1* gene in K-9 was identical to that in strain K-7. We found that the *PRO1* gene sequence in K-9-AZC included a mixture of G and T at nucleotide position 237, leading to amino acid replacement of Gln to His at position 79 in the amino acid sequence of GK. This mutation has not been reported. Glutamine at position 79 in the kinase domain is a highly conserved residue among various GKs in microorganisms (Fig. 1a).

Here, we focused only on the *PRO1* allele. However, the possibility of mutations in the other genes related to proline metabolism and cellular transport should not be ignored. Hence, it would be worthwhile to determine the whole genome sequence of K-9-AZC.

### Enzymatic analysis of the Q79H variant GK

So far, no study has reported on the function of Gln79 in the binding of substrates and/or its sensitivity to feedback inhibition by proline. Thus, to investigate the effect of the amino acid substitution of Gln79 to His on feedback inhibition by proline, we further analyzed the enzymatic activity of the Q79H variant GK using the purified recombinant protein. We also tested the enzymatic activity of the I150T variant GK, which is greatly insensitive to feedback inhibition, leading to proline accumulation [15]. As shown in Table 2, the specific activity of GK in the Q79H variant was slightly reduced compared with the wild-type (WT) and I150T variant GKs. When the enzyme assay was done in the presence of proline, the GK activity of WT was markedly inhibited, in agreement with previous results [15]. Interestingly, we found that the relative activity of the Q79H variant was 96% in the presence of 100 mM proline (Fig. 1b), which was much higher than that of the I50T variant (59%). The level of activity in the Q79H variant was still 87% even in the presence of 400 mM proline (data not shown). These results indicate that the Q79H variant was highly desensitized to feedback inhibition by proline, suggesting that this diminished sensitivity to proline feedback inhibition in GK causes proline oversynthesis in yeast cells.

**Table 2:** Specific activities of the recombinant GKs

Enzyme	Specific activity (U/mg)^a^
WT	14.3 ± 0.381^b^
Q79H	11.0 ± 0.605^b^
I150T	14.3

^a^The specific activity was determined as described in the “Materials and methods” section

^b^The values are the means and standard deviations of results from three independent experiments

Previous studies [9, 13–15] reported that a region between the hypothetical β-sheet (Ile136-Asn140) and α-helix (Asn155-Ile166) is important for the binding to glutamate in *E. coli* and for the sensitivity to feedback inhibition by proline in both *E. coli* and *S. cerevisiae*, which were situated in the loop between β4 and αE in the *E. coli* GK (Fig. 1a) [9]. It appears that Gln73, which corresponds to Gln79 in the *S. cerevisiae* GK, does not directly interact with the substrate and inhibitor in the crystal structure of the *E. coli* GK [9]. Although the function of Gln79 is not clear, the amino acid replacement of Gln79 by a bulkier residue, like histidine, might alter the local conformation of GK to lower the affinity to proline or to inhibit the binding of some group(s) to proline. Future studies will elucidate the function of Gln79 and obtain knowledge applicable to the enzyme engineering of GK for higher production of proline.

### Effects of the Q79H variant GK on the fermentation and metabolite profiles in sake mash

**Fig. 2:**
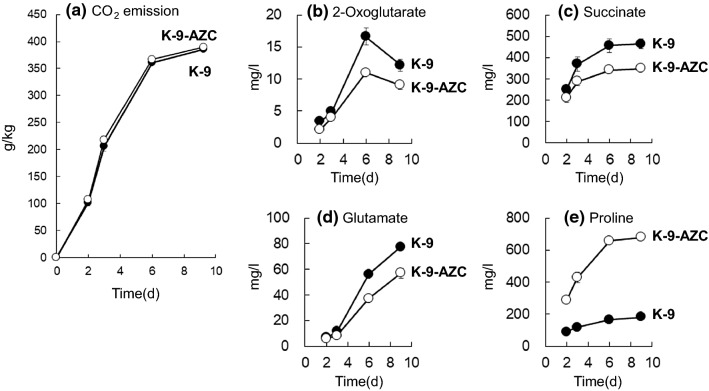
Concentrations of supernatant organic acids and amino acids in sake mash. Comparison of **a** CO_2_ emission, **b** 2-oxoglutarate, **c** succinate, **d** glutamate, and **e** proline between K-9 (closed circles) and K-9-AZC (open circles). The values are the means and standard deviations of results from three independent experiments

We next brewed laboratory scale sake with sake yeast strains and analyzed general compounds in sake mash. The time point of sampling was days 2, 3, 6, and 9. Days 2 and 3 present the early and middle stage of proline production, respectively. Also, both days 6 and 9 indicate the late stage of proline production. As shown in Fig. 2a, there was no significant difference between strains K-9 and K-9-AZC with regard to fermentation speed. Table 3 shows that K-9-AZC produced ethanol at the same rate as K-9, whereas acid levels were lower in sake brewed with K-9-AZC (1.38 ± 0.03) than those in sake brewed with K-9 (1.71 ± 0.06). In addition, during fermentation, it appears that 2-oxoglutarate and succinate levels in sake brewed with K-9-AZC were lower than those in sake brewed with K-9 (Fig. 2b, c), probably due to the low acid levels obtained from K-9-AZC (Table 3). In contrast, sake brewed with K-9-AZC contained four times more proline and ~ 26% less glutamate than did the sake brewed with K-9 (Table 3). This is probably because the Q79H variant GK is insensitive to proline feedback inhibition, leading to intracellular proline accumulation. These results were similar to our recent findings [7]. Together, the present and previous results suggested that the succinate content is influenced by enhanced proline synthetic activity in K-9-AZC, because glutamate, which is converted into proline, is also supplied from 2-oxoglutarate. As shown in Fig. 2 and Table 3, both 2-oxoglutarate and glutamate concentrations in K-9-AZC were lower than those in K-9, leading to a lower concentration of succinate in K-9-AZC.

**Table 3:** Fermentation profiles in brewed sake with K-9 and K-9-AZC

Parameter	Sake yeast strain	
	K-9	K-9-AZC
Sake meter	21.8 ± 0.3	22.1 ± 0.2
Ethanol (vol%)	14.8 ± 0.0	14.8 ± 0.0
Acidity	1.71 ± 0.06	1.38 ± 0.03
Amino acid content	1.00 ± 0.02	1.06 ± 0.01
Organic acid (mg/l)		
Citrate	66 ± 1	69 ± 1
2-Oxoglutarate	12 ± 1	9 ± 1
Succinate	464 ± 23	349 ± 11
Fumarate	2 ± 0	2 ± 0
Malate	134 ± 3	141 ± 3
Amino acid (mg/l)		
Glutamate	77 ± 2	57 ± 4
Proline	182 ± 8	679 ± 7

The values are the means and standard deviations of results from three independent experiments

**Fig. 3:**
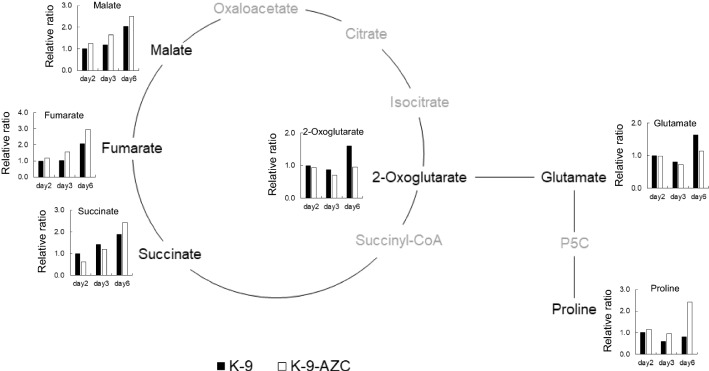
Relative ratio of intracellular organic acids and amino acids between K-9 (closed bars) and K-9-AZC (open bars). Yeast cells were recovered from above-mentioned sake mash. Relative ratio is expressed as fold changes relative to the concentrations of those in K-9 at day 2. P5C; Δ^1^-pyrroline-5-carboxylate

We also measured various intracellular metabolites in the above-mentioned sake mash (Fig. 3). The time point of sampling was days 2, 3 and 6, because extracellular proline and succinate concentrations were maintained after days 6 and 9, based on our preliminary experiment (data not shown). At day 6, relative ratio of intracellular 2-oxoglutarate and glutamate in K-9-AZC was lower than that in K-9. In contrast, relative ratio of proline in K-9-AZC was higher than that in K-9. These results suggest that both 2-oxoglutarate and glutamate were utilized for overproduction of proline. On the other hand, relative ratio of intracellular succinate in K-9-AZC was less than that in K-9 on days 2 and 3 (62% and 84%, respectively), but was ~ 30% higher than K-9 on day 6. Additionally, malate and fumarate were also accumulated in K-9-AZC. The succinate concentration at the early stage of fermentation (days 2 and 3) might be affected by a low concentration of 2-oxoglutarate. However, the reductive TCA pathway is considered to proceed at the late stage of fermentation, because it was unaffected by glutamate [3]. It was previously suggested that fumarate reductase in the reductive TCA pathway provides the only way to regenerate the prosthetic group FAD/FMN of flavin enzymes under anaerobic conditions for the control of the intracellular redox balance [1, 2, 4]. Furthermore, FAD is necessary for the proline oxidase Put1, which oxidizes proline to Δ^1^-pyrroline-5-carboxylate as a cofactor.

**Fig. 4:**
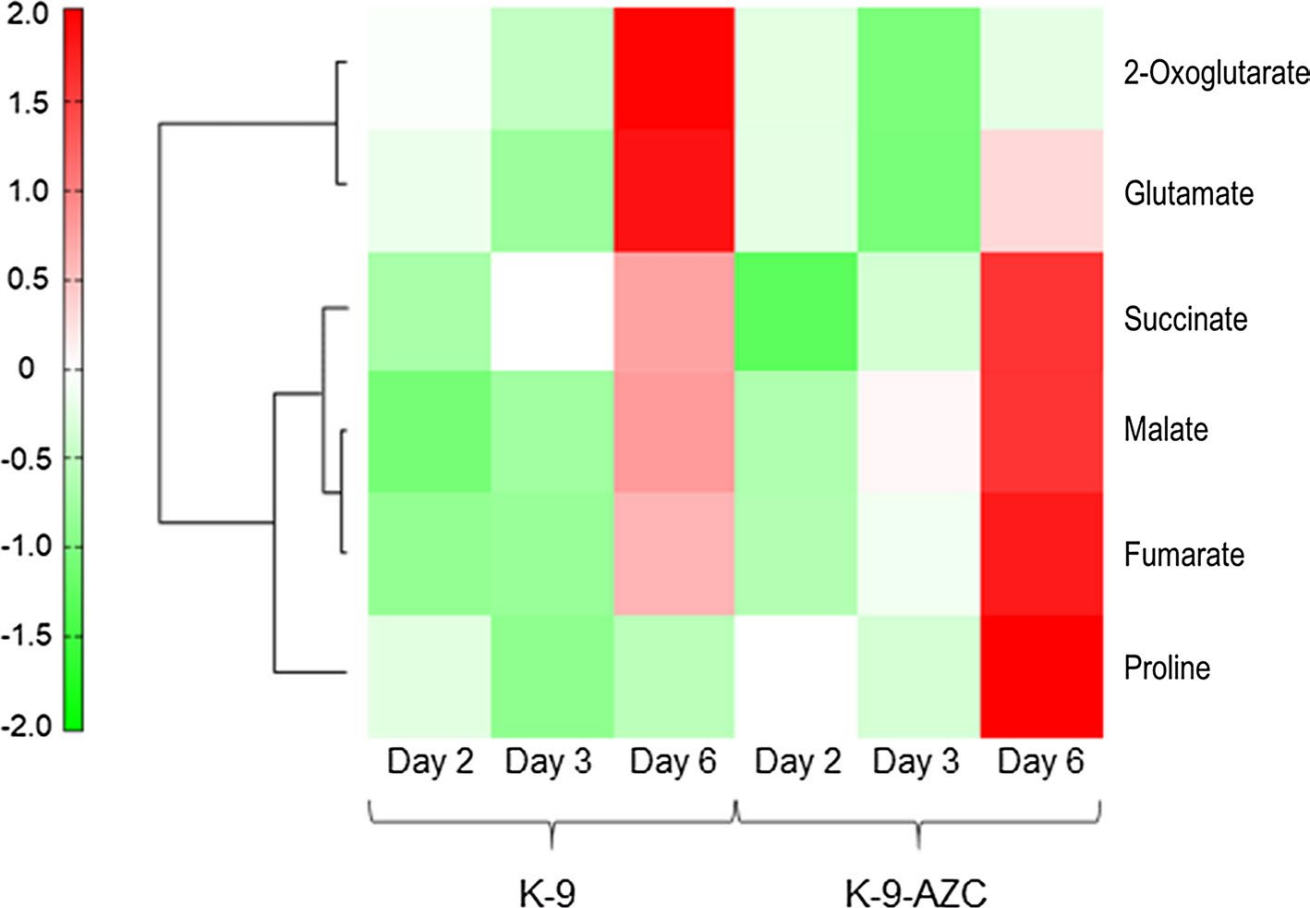
Hierarchical cluster analysis (HCA) of 2-oxoglutarate, succinate, fumarate, malate, glutamate and proline excerpted from the 258 intracellular metabolites profiles obtained from CE-TOFMS analysis. The cells and sampling times were colored by the normalized intensities. Both the compounds (rows) and the samples (columns) were clustered and reordered by the similarity of the intensity patterns. Red or green color refers to higher or lower value, respectively, compared to the average. Longitudinal or horizontal axis indicates compounds or samples, respectively

In addition, we performed HCA of the 258 intracellular metabolites profiles obtained from CE-TOFMS analysis (Fig. 4). The result suggests that the correlation between succinate and the group of malate and fumarate is higher than that between the group of 2-oxoglutarate and glutamate. Accordingly, intracellular succinate could be supplemented mainly via the reductive TCA pathway in K-9-AZC. Nevertheless, it is still unclear the reason why succinate content in sake brewed with K-9-AZC is lower than that in sake brewed with K-9 (Table 3). A *kgd1* mutant, in which the mitochondrial 2-oxoglutarate dehydrogenase is deficient, displayed lower levels of extracellular succinate production on glutamate as a sole nitrogen source compared with the wild-type strain [3]. Thus, extracellular succinate might be produced mainly via the oxidative TCA pathway, suggesting that succinate production in K-9-AZC was lower than that in K-9 during anaerobic fermentation.

We previously constructed a haploid sake yeast strain by replacing the wild-type *PRO1* allele with the *PRO1*^D154N^ allele [23]. The resultant strain accumulated proline and was more tolerant to ethanol stress than the control strain. We also found that the fermentation profile of sake brewed with this proline-accumulating strain is not markedly influenced, but that the strain contains fivefold more proline than the control strain. We also constructed self-cloning diploid sake yeast strains that accumulate proline by replacing the wild-type *PRO1* allele with the *PRO1*^D154N^ or *PRO1*^I150T^ allele [22]. Sake brewed with the proline-accumulating strains contained two- to threefold more proline than sake brewed with the parent strain. It was also suggested that intracellular proline does not affect overall fermentation profiles, but reduces fermentation time in terms of the ethanol production rate. Although self-cloning yeasts, which do not contain any foreign genes or DNA sequences except for yeast DNA, do not have to be treated as genetically modified yeasts, the conventional methods for breeding sake yeasts are more acceptable to consumers than is the use of self-cloning yeasts. Thus, the approach described here could be a practical method for breeding novel sake yeast strains.

Proline may impart sake with some sensory qualities. In particular, high levels of proline may contribute to its perceived sweetness. On the other hand, the taste of succinic acid is sour and umami. Based on these characteristics of proline and succinate, sake brewed with K-9-AZC could be promising to improve the taste of light-bodied sake.

## Conclusions

The present study is the first to report an analysis of diploid sake yeast mutant K-9-AZC, which increases proline and decreases succinate in sake. In addition, we found that a novel *PRO1* mutation (*PRO1*^Q79H^) in sake yeast desensitized the proline feedback inhibition of GK, leading to intracellular proline accumulation. We believe that not only sake yeast but also other brewing yeasts (e.g., those used to make beer, wine, and shochu) with higher proline and lower succinate productivities could contribute to the diversity of taste in alcoholic beverages.

## References

[1] Arikawa Y, Enomoto K, Muratsubaki H, Okazaki M (1998). Soluble fumarate reductase isoenzymes from *Saccharomyces cerevisiae* are required for anaerobic growth. FEMS Microbiol.

[2] Camarasa C, Faucet V, Dquin S (2007). Role in anaerobiosis of the isoenzymes for *Saccharomyces cerevisiae* fumarate reductase encoded by *OSM1* and *FRDS1*. Yeast.

[3] Camarasa C, Grivet JP, Dequin S (2003). Investigation by ^13^C-NMR and tricarboxylic acid (TCA) deletion mutant analysis of pathways for succinate formation in *Saccharomyces cerevisiae* during anaerobic fermentation. Microbiol.

[4] Enomoto K, Arikawa Y, Muratsubaki H (2002). Physiological role of soluble fumarate reductase in redox balancing during anaerobiosis in *Saccharomyces cerevisiae*. FEMS Microbiol.

[5] Kaino T, Tasaka Y, Tatehashi Y, Takagi H (2012). Functional analysis of the C-terminal region of γ-glutamyl kinase of *Saccharomyces cerevisiae*. Biosci Biotechnol Biochem.

[6] Kitagaki H, Kitamoto K (2013). Breeding research on sake yeasts in Japan: history, recent technological advances, and future perspectives. Annu Rev Food Sci Tecnol..

[7] Kotaka A, Nakamura Y, Kasai H, Watanabe Y, Morimoto-Sakakibara M, Matsumura K, Hata Y (2019). Development of amino-acid-analogue-resistant and low acid-producing sake yeast for commercial scale sake brewing. J Brew Soc Jpn.

[8] Ljungdahl PO, Daignan-Fornier B (2012). Regulation of amino acid, nucleotide, and phosphate metabolism in *Saccharomyces cerevisiae*. Genetics.

[9] Marco-Marín C, Gil-Ortiz F, Pérez-Arellano I (2007). A novel two-domain architecture within the amino acid kinase enzyme family revealed by the crystal structure of *Escherichia coli* glutamate 5-kinase. J Mol Biol.

[10] Morita Y, Nakamori S, Takagi H (2003). l-Proline accumulation and freeze tolerance in *Saccharomyces cerevisiae* are caused by a mutation in the *PRO1* gene encoding γ-glutamyl kinase. Appl Eviron Microbiol.

[11] Negoro H, Kotaka A, Matsumura K, Tsutsumi H, Hata Y (2016). Enhancement of malate-production and increase in sensitivity to dimethyl succinate by mutation of the *VID24* gene in *Saccharomyces cerevisiae*. J Biosci Bioeng.

[12] Okazaki N (1993) Seishu, Gousei-seishu, p. 7–33, In: Nishiya N (ed), The annotation of the official methods of analysis of the National Tax Administration Agency, Japan, 4th ed., The Brewing Society of Japan, Tokyo. **(in Japanese)**

[13] Pérez-Arellano I, Rubio V, Cervera J (2006). Mapping active site residues in glutamate-5-kinase. The substrate glutamate and the feed-back inhibitor proline bind at overlapping sites. FEBS Lett.

[14] Pérez-Arellano I, Rubio V, Cervera J (2010). Molecular mechanisms modulating glutamate kinase activity. Identification of the proline feedback inhibitor binding site. FEBS Lett.

[15] Sekine T, Kawaguchi A, Hamano Y, Takagi H (2007). Desensitization of feedback inhibition of the *Saccharomyces cerevisiae* γ-glutamyl kinase enhances proline accumulation and freezing tolerance. Appl Eviron Microbiol.

[16] Sienkiewicz N, Ong HB, Fairlamb AH (2018). Characterisation of a putative glutamate 5-kinase from *Leishmania donovani*. FEBS J.

[17] Soga T, Heiger DN (2000). Amino acid analysis by capillary electrophoresis electrospray ionization mass spectrometry. Anal Chem.

[18] Soga T, Ohashi Y, Ueno Y, Naraoka H, Tomita M, Nishioka T (2003). Quantitative metabolome analysis using capillary electrophoresis mass spectrometry. J Proteome Res.

[19] Soga T, Ueno Y, Naraoka H, Ohashi Y, Tomita M, Nishioka T (2002). Simultaneous determination of anionic intermediates for *Bacillus subtilis* metabolic pathways by capillary electrophoresis electrospray ionization mass spectrometry. Anal Chem.

[20] Sugimoto M, Wong DT, Hirayama A, Soga T, Tomita M (2010). Capillary electrophoresis mass spectrometry-based saliva metabolomics identified oral, breast and pancreatic cancer-specific profiles. Metabolomics.

[21] Takagi H (2019). Metabolic regulatory mechanisms and physiological roles of functional amino acids and their applications in yeast. Biosci Biotechnol Biochem.

[22] Takagi H, Matsui F, Kawaguchi A, Wu H, Shimoi H, Kubo Y (2007). Construction and analysis of self-cloning sake yeasts that accumulate proline. J Biosci Bioeng.

[23] Takagi H, Takaoka M, Kawaguchi A, Kubo Y (2005). Effect of l-proline on sake brewing and ethanol stress in *Saccharomyces cerevisiae*. Appl Environ Microbiol.

[24] Terao Y, Nakamori S, Takagi H (2003). Gene dosage effect of l-proline biosynthetic enzymes on l-proline accumulation and freeze tolerance in *Saccharomyces cerevisiae*. Appl Eviron Microbiol.

[25] Wampler DE, Westhead EW (1968). Two aspartokinases from *Escherichia coli*. Nature of the inhibition and molecular changes accompanying reversible inactivation. Biochemistry.

